# Real-World Comparative Evaluation of Add-On Glucagon-like Peptide 1 Receptor Agonist in Type 2 Diabetes Treated with or without Insulin

**DOI:** 10.3390/ph15121569

**Published:** 2022-12-15

**Authors:** Hsuan-Wen Chou, Kai-Pi Cheng, An-Chi Lin, Hao-Chang Hung, Ching-Han Lin, Chih-Chen Wang, Hung-Tsung Wu, Horng-Yih Ou

**Affiliations:** 1 Department of Internal Medicine, National Cheng Kung University Hospital, College of Medicine, National Cheng Kung University, Tainan 704302, Taiwan; 2 Division of Endocrinology and Metabolism, Department of Internal Medicine, Kaohsiung Veterans General Hospital, Kaohsiung 813414, Taiwan; 3 Department of Internal Medicine, National Cheng Kung University Hospital Dou-Liou Branch, College of Medicine, National Cheng Kung University, Douliu 640003, Taiwan; 4 Department of Internal Medicine, School of Medicine, College of Medicine, National Cheng Kung University, Tainan 701401, Taiwan

**Keywords:** diabetes, Glucagon-like peptide 1 receptor agonists, glycemic control, insulin, real world study

## Abstract

Glucagon-like peptide 1 receptor agonist (GLP-1 RA) is a potent antidiabetic agent with cardiorenal and weight-losing benefits in patients with type 2 diabetes (T2D). The combination of GLP-1 RA with basal insulin has been suggested in several clinical studies as a useful treatment for intensifying insulin therapy in T2D. However, there has been no real-world evidence study comparing the glycemic effects of GLP-1 RAs add-on to background treatment with and without insulin. A retrospective study was performed in 358 patients with T2D who initiated liraglutide or dulaglutide. Among them, 147 patients were prior and concurrent insulin users, and 211 patients were non-insulin users. After 12 months of GLP-1 RA treatment, the changes in hemoglobin A1c (HbA1C) and body weight were evaluated. The effectiveness of GLP-1 RAs on HbA1C reduction was greater in insulin users than non-insulin users at 12 months (−1.17% vs. −0.76%; *p* = 0.018). There was no significant difference in body weight change between insulin users and non-insulin users at 12 months (−1.42 kg vs. −1.87 kg; *p* = 0.287). The proportion of responders (decrease of HbA1C > 1%) in insulin users was much higher than that in non-insulin users (48% vs. 37 %; *p* = 0.04). In insulin users, those who had increased insulin dosage at 12 months had significantly less HbA1C reduction than that of non-increased patients (−0.62% vs. −1.57%; *p* = 0.001). GLP-1 RAs provide superior glucose-lowering effects in insulin-treated patients compared with non-insulin-treated patients with T2D without significant differences in body weight decrease.

## 1. Introduction

Type 2 diabetes mellitus (T2D) has become an important health issue worldwide since the number of people with diabetes is increasing gradually [1]. The total number of people with diabetes is estimated to rise from 537 million in 2021 to 783 million by 2040 [2]. Inadequately controlled hyperglycemia leads to both micro- and macrovascular complications, which negatively impact the quality of life of people with diabetes and increase the economic burden of public healthcare systems. 

In recent years, many new antidiabetic agents have been developed targeting different metabolic pathways involved in T2D [3]. Currently, two different injectable therapies are available for diabetes, one is insulin and the other is glucagon-like peptide-1 receptor agonist (GLP-1 RA), which is a relatively new class of glucose-lowering medications. In randomized controlled trials, GLP-1 RAs increase glucose-stimulated insulin secretion and slow gastric emptying to achieve glycemic control and weight management. GLP-1 RAs also suppress postprandial glucagon secretion to reduce hyperglycemia [4]. In addition, GLP-1 RAs have beneficial effects on cardiovascular and renal outcomes as well as decreasing mortality in patients with T2D [5,6,7,8,9]. Therefore, when injectable therapy is needed, most clinical practice recommendations, including the very recent American Diabetes Association guideline in 2022, suggest GLP-1 RA is preferred to insulin based on its additional benefits in body weight and organ protection [10]. However, due to its effect on slowing gastric emptying, gastrointestinal side effects, such as nausea and vomiting, were reported in many GLP-1 RA studies [11]. GLP-1 RA may also slightly increase the risk of gallbladder disease [12]. 

Despite not being recommended, insulin is still commonly prescribed as the first injectable therapy prior to GLP-1 RAs for inadequately controlled diabetes in real-world practice owing to its cost-effectiveness. Although the positive effects of GLP-1 RAs add-on therapy to insulin have been demonstrated in many studies [13,14,15,16], there has been no study comparing the glycemic effects of GLP-1 RAs add-on to background treatment with and without insulin. Adding GLP-1 RAs to insulin improved glycemic control with similar gastrointestinal side effects without significant change in weight or daily insulin dose in one study of retrospective chart reviews [17]. Furthermore, this combination has less weight gain and hypoglycemia than insulin treatment alone because of the weight-losing effects of GLP-1 RAs and usually less insulin dosage needed to achieve better glycemic control compared with insulin treatment alone. Thus, in this retrospective study, we aimed to investigate the effects of GLP-1 RAs in insulin-treated and non-insulin-treated patients with T2D in a real-world setting.

## 2. Results

We enrolled 358 consecutive patients in this study. The patients were divided into two groups based on the presence or absence of prior and concurrent insulin treatment. Among them, 147 patients were insulin users, and 211 patients were non-insulin users. The baseline characteristics of the study subjects are shown in Table 1. Insulin users were older (56.7± 13.1 vs. 50.6 ± 12.9 years; *p* < 0.001), had longer diabetes duration (12.1 ± 7.1 vs. 8.7 ± 6.5 years; *p* < 0.001), and had higher HbA1C (9.50 ± 1.70 vs. 8.84 ± 1.60 %; *p* < 0.001), whereas body mass index (BMI) was lower (29.4 ± 5.2 vs. 31.3 ± 5.3; *p* = 0.001). The proportion of patients with an eGFR < 45 mL/min/1.73 m^2^ in the insulin group was higher than in the non-insulin group (16% vs. 6%; *p* = 0.002). In addition, the insulin group was treated less often with metformin (72% vs. 91%; *p* < 0.001) and insulin secretagogues (42% vs. 72%; *p* < 0.001) than the non-insulin group.

**Figure 1:**
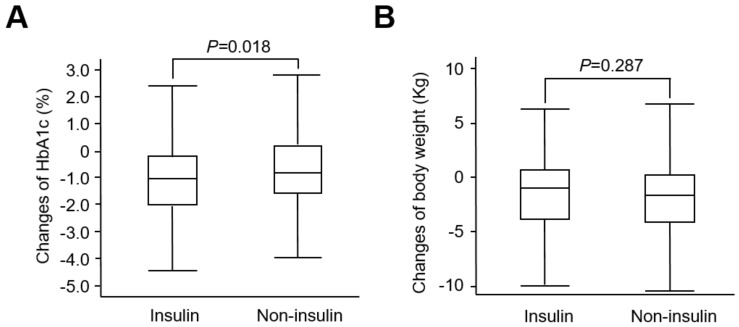
The comparisons of HbA1C (**A**) and body weight (**B**) changes from baseline to 12 months between the insulin and non-insulin users.

As for glycemic control, we found that GLP-1 RAs had a greater effect on HbA1C reductions in insulin users than non-insulin users at 12 months (−1.17% vs. −0.76%; *p* = 0.018) (Figure 1A). This difference remains statistically significant even after adjustment for age, diabetes duration, BMI, and eGFR. In contrast, there was significant weight loss in both insulin users (−1.42 kg) and non-insulin users (−1.87 kg) at 12 months (Figure 1B). This weight-loss effect showed no differences between the two groups after the treatment of GLP-1 RA (*p* = 0.287). When calculating patients treated with liraglutide (*n* = 217) or dulaglutide (*n* = 141) separately, we found a similar trend in both insulin users and non-insulin users of these two GLP-1 RAs. This justifies combining liraglutide and dulaglutide as “GLP-1 RA” in our study. In contrast, in patients who initiated insulin during the same period (*n* = 20, age 53.4 ± 12.3; M/F, 10/10; diabetes duration 10.7 ± 7.2), the HbA1C improved significantly after insulin treatment (9.40 ± 1.90 % to 8.20 ± 1.30 %, *p* = 0.01), but was accompanied with increased body weight (72.8 ± 14.2 to 75.6 ± 14.0, *p* = 0.02). These results are compatible with current knowledge that insulin has glucose-lowering effects at the expense of gaining weight.

**Figure 2:**
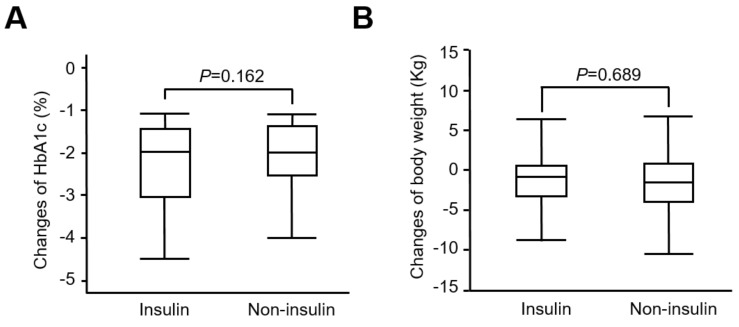
The comparisons of HbA1C (**A**) and body weight (**B**) changes of responders from baseline to 12 months between the insulin and non-insulin groups.

In addition, we further defined an HbA1C reduction of >1% at 12 months as GLP-1 RAs responders. The proportion of responders in insulin users was much higher than that in non-insulin groups (48% vs. 37 %; *p* = 0.04). However, there were no differences in HbA1C change (−2.46% vs. −2.17%, *p* = 0.162) (Figure 2A) and body weight change (−1.11 kg vs. −1.37 kg; *p* = 0.689) (Figure 2B) between responders of the insulin and non-insulin groups. Further, there were no severe hypoglycemia events or major adverse effects noted in these patients.

Finally, to explore the potential effect of changes in insulin dosage on glycemic and body weight in insulin users, we divided the study subjects by the changes in insulin dosage at the 12th month. The increased dosage group (*n* = 63, 43%) was defined by an insulin-dosage increase of ≥ 0 IU at the 12th month and all the others (with unchanged or decreased dosage) were designated as the non-increased group (*n* = 84, 57%). We found both increased and non-increased groups had comparable fasting plasma glucose, HbA1C, diabetes duration, and body weight at baseline. As a whole, the total daily insulin dose (TDD) was not changed in insulin-treated subjects (mean TDD of 35.0 IU vs. 35.7 IU, 0 months vs. 12 months; *p* = 0.6). Interestingly, we noted that the increased group had a significantly less HbA1C reduction than that of the non-increased group (−0.62% vs. −1.57%; *p* = 0.001). However, there was no significant difference in body weight change between the increased group and the non-increased group (−2.1 kg vs. −0.95 kg; *p* = 0.06).

During the study period, one patient in the “insulin use” and two patients in the “non-insulin use” group developed acute coronary syndrome (*p* = NS). No gallbladder disease event occurred in all of the subjects. We also observed that the urinary albumin-to-creatinine ratio (UACR) decreased significantly in both “insulin use” (604 ± 1136 to 462 ± 1123, *p* = 0.04) and “non-insulin use” groups (274 ± 747 to 205 ± 676, *p* = 0.047), indicating renal benefits of GLP-1 RAs with/without insulin.

## 3. Discussion

To the best of our knowledge, this is the first real-world retrospective study investigating the glycemic effects of GLP-1 RAs add-ons to background treatment with or without insulin in patients with T2D. We found that GLP-1 RAs had a greater reduction in HbA1C in insulin-treated patients than in non-insulin-treated ones. Our finding not only confirmed previous studies [13,16] showing that irrespective of the baseline HbA1C, GLP-1 RAs could provide additional glucose-lowering effects besides insulin but also suggested that the combination of these two injectable therapies could further benefit glycemic control than GLP-1 RAs alone without the increment in body weight.

GLP-1 RAs have an important role in current diabetes treatment. The very recent ADA/EASD guideline [10,18] suggests that in patients with T2D with established or having a high risk for atherosclerotic cardiovascular disease and chronic kidney disease, GLP-1 RAs with demonstrated cardiovascular benefit are recommended, even independent of baseline HbA1C. For glycemic control, when injectable therapy is needed, GLP-1 RAs are the preferred option to insulin since the risk of hypoglycemia and body weight gain are less than insulin therapy. Additionally, if basal insulin has been adjusted to an acceptable fasting blood glucose level but HbA1C does not achieve the goal (as shown in our reference group of patients), adding on prandial insulins or GLP-1 RAs for those who have not used it should be considered [10]. As compared with adding on prandial insulin, combining GLP-1 RAs with basal insulin is more potent in lowering glycemia and reducing weight gain and hypoglycemic events. Our study further suggests that adding on GLP-1 RAs in insulin-treated patients provides greater glycemic efficacy compared with non-insulin-treated patients. 

The mechanisms of greater glycemic efficacy observed in insulin-treated patients may be several folds. First, the combination of GLP-1 RAs with basal insulin targets both fasting and, more importantly, postprandial hyperglycemia [19]. It is noteworthy that as the disease progresses, progressive loss of beta-cell mass and function in patients with T2D, irrespective of glucose-lowering treatment, was demonstrated in the United Kingdom Prospective Diabetes Study [20]. What is even worse is that chronic hyperglycemia causes glucose toxicity, which leads to beta-cell failure [21]. Thus, it is plausible to initiate insulin therapy in patients with diabetes of long duration and suboptimal glycemic control, as shown in the current study (mean duration 8.7–12.1 years, mean HbA1C 8.84–9.50%). 

In addition, timely treatment with insulin also allows pancreatic beta-cell rest and helps preserve beta-cell function [22]. On the other hand, therapies that stimulate insulin secretion (such as sulfonylurea) increase the workload of beta-cells and thus cause beta-cell failure over time [23,24], which presumably makes GLP-1 RAs less effective. This notion is supported by previous studies showing GLP-1 RAs have more favorable effects if they are used at an early stage of diabetes [25,26]. In the current study, given the longer diabetes duration and higher glycemia in insulin-treated subjects, prior insulin treatment probably rests the beta-cell to facilitate the glycemic effects of GLP-1 RAs. 

In addition, we found, in the insulin-treated group, the significant HbA1C reduction was not attributed to the increased insulin dose since the TDD was not changed between the baseline and the 12th month. Paradoxically, those who increased insulin dosage at the end of the study had less HbA1C decrease (mean difference 0.95%) than the non-increased group. This finding indicates that patients in the increased group did not respond well to GLP-1 RA therapy so insulin doses were up-titrated. Our findings are consistent with a meta-analysis of randomized controlled trials, revealing that patients with a good response to GLP-1 RA do not exceed 50–60% [27]. Regarding this, although some previous studies aimed to find predictors of good response [28,29,30], the results have been controversial. Some suggested patients with a higher pre-treatment glycemia had more glucose-lowering response [28], but others demonstrated higher BMI, and higher mean pre-prandial blood glucose was associated with less response [29]. 

In our study, the significantly decreased UCAR in GLP-1 Ras-treated patients (with or without insulin) confirmed the renal benefits of GLP-1 RAs. Only three patients developed cardiovascular events during the observation period, which is suggestive of the cardiovascular safety of GLP-1 RAs.

There are some limitations in this work. First, as a real-world retrospective study, some baseline characteristics were not comparable between the two study groups. Therefore, we should be careful in interpreting the results. However, after adjustment for covariates, we were still able to demonstrate the significant difference in glycemic control between the groups. Second, since this was a single-center study and the sample size was relatively small, a larger sample could be more readily generalized to the population in the future. Third, only two GLP-1 RAs were analyzed in this study; it remains unclear whether this is a class effect so that newer GLP-1 RAs, such as semaglutide, in either injectable or oral form, have a similar effect. Furthermore, adherence data, which is an important factor affecting the treatment results, are not available in our study. With regard to this issue, nearly 90% of the patients have been enrolled in the structured and integrated “Diabetes Shared Care Program” provided by physicians and certified diabetes educators to ensure compliance. Finally, the findings in our study may not be generalized to other ethnicities since East Asian T2D patients have distinct characteristics, such as early loss of beta-cell function with reduced insulin secretion, compared with Caucasians [5].

## 4. Material and Methods

The present study was designed as a retrospective observational study. The study protocol was approved by the Human Experiment and Ethics Committee of National Cheng Kung University Hospital (NCKUH) (Tainan, Taiwan) (A-ER-108-321, approved on 5 November 2019). Consecutive patients with T2D aged >20 years old who started GLP-1 RA (liraglutide or dulaglutide) between 1 September 2013, and 31 December 2019 (index date) and regularly followed up at the endocrinology out-patient clinic of NCKUH were enrolled. In addition, we retrospectively collected 20 patients with diabetes that initiated insulin during the same period for comparison of the glycemic and weight effects.

We excluded the subjects with: 1. a history of pancreatitis; 2. acute infections; 3. active malignant diseases; 4. pregnancy; and 5. who have taken steroids for over seven days. Those who did not follow up within 12 months after GLP-1 RA treatment were also excluded. 

We collected anthropometric parameters, including body height (to the nearest 0.1 cm), body weight (to the nearest 0.1 kg), and BMI (in kg/m^2^), calculated as weight (in kilograms) divided by body height (in meters) squared [31].

All of the patients fasted overnight for at least 12 h before blood sampling. The fasting plasma glucose, creatinine, alanine aminotransferase, and lipid profiles were measured with an autoanalyzer (Hitachi 747E; Tokyo, Japan) at the central laboratory of NCKUH. HbA1c was determined using Tosoh Automated Glycohemoglobin Analyzer (Tokyo, Japan). Urinary protein excretion was assessed by urine albumin/creatinine ratio. The modification of diet in the renal disease equation was used to calculate the estimated glomerular filtration rate (eGFR, mL/min/1.73 m^2^). Events of severe hypoglycemia, defined as a severe event where assistance for the treatment of hypoglycemia is needed [32], and changes in the antihyperglycemic regimen (including oral antidiabetic drugs and/or insulin) during the study period were retrospectively obtained. 

To detect a difference in HbA1C changes of 0.4% (−0.6% vs. −1.0%) with a ratio of non-insulin to insulin groups of 1.5, we would need 219/131 patients in the non-insulin/insulin group to achieve a power of 80% and a level of significance of 5%.

For the data analysis, the Statistical Package for the Social Sciences (SPSS version 21.0; Chicago, IL, USA) was used. Clinical characteristics were expressed as mean ± standard deviation (SD) for continuous variables and as percentages for categorical variables. To analyze the differences in categorical and continuous variables between groups, Chi-square and Student’s *t*-tests were used, respectively. We considered a *p*-value of less than 0.05 as statistically significant.

## 5. Conclusions

Our study indicates that GLP-1 RAs can be preferentially combined with basal insulin. GLP-1 RAs provide superior glucose-lowering effects in insulin-treated patients compared with non-insulin-treated patients with T2D, without significant differences in body weight decrease. Therefore, we suggest GLP-1 RAs as a good choice for those who have not achieved glycemic goals even after initiating insulin.

## Figures and Tables

**Table 1:** Comparisons of baseline clinical parameters between insulin users and non-insulin users.

	Insulin User	Non-Insulin User	*p*-Value (Insulin User vs. Non-Insulin User)
Number	147	211	
Age (years)	56.7 ± 13.1	50.6 ± 12.9	<0.001
Male (%)	51	51	NS
DM duration (years)	12.1 ± 7.1	8.7 ± 6.5	<0.001
BW (kg)	77.2± 16.0	83.7 ± 16.1	<0.001
BMI (kg/m^2^)	29.4 ± 5.2	31.3± 5.3	0.001
SBP (mmHg)	136 ± 18	135 ± 16	NS
DBP (mmHg)	81 ± 10	82 ± 10	NS
Enrollment into “Diabetes Shared Care Program” (%)	86	91	NS
Comorbidities			
Cerebrovascular disease (%)	19	17	NS
Coronary artery disease (%)	19	17	NS
Stroke (%)	11	9	NS
Peripheral artery disease (%)	0	1	NS
Congestive heart failure (%)	3	7	NS
Biochemical data			
Fasting plasma glucose (mg/dL)	186.01 ± 72.88	174.98 ± 55.87	0.12
A1C (%)	9.50 ± 1.70	8.84 ± 1.60	<0.001
Creatinine (mg/dL)	1.15 ± 1.60	0.83 ± 0.43	0.007
Estimated GFR (ml/min/1.73 m^2^) < 45 (%)	16	6	0.002
ALT (U/L)	36 ± 28	43 ± 26	0.017
Urinary albumin-to-creatinine ratio (mg/g)	604 ± 1136	274 ± 747	0.001
Total cholesterol (mg/dL)	158.8 ± 38.2	167.0 ± 42.8	NS
Triglyceride (mg/dL)	191.8 ± 207.8	203.2 ± 164.1	NS
HDL-C (mg/dL)	44.6 ± 13.6	44.0 ± 11.6	NS
LDL-C (mg/dL)	95.6 ± 32.34	98.3 ± 32.4	NS
Other anti-diabetes medications			
Metformin (%)	72	91	<0.001
Insulin secretagogues (%)	42	72	<0.001
Pioglitazone (%)	14	12	NS
Acarbose (%)	10	8	NS
DPP-4 inhibitor (%)	1	3	NS
SGLT-2 inhibitor (%)	5	8	NS

Data are expressed as the mean ± standard deviation (SD) or as a percentage. BW, body weight; BMI, body mass index; SBP, systolic blood pressure; DBP, diastolic blood pressure; CVD, cardiovascular disease; MI, myocardial infarction; CAD, coronary artery disease; PAOD, peripheral arterial occlusive disease; CHF, congestive heart failure; FPG, fasting plasma glucose; A1C, hemoglobin A1c; eGFR, estimated glomerular filtration rate; ALT, alanine aminotransferase; UACR, urine albumin creatinine ratio; HDL-C, high-density lipoprotein cholesterol; LDL-C, low-density lipoprotein cholesterol; DPP-4 inhibitor, Dipeptidyl peptidase-4 inhibitor; SGLT-2 inhibitor, Sodium-glucose co-transporter-2 inhibitor.

## Data Availability

Data is contained within the article.
